# Comparative analysis of regulations and studies on stem cell therapies: focusing on induced pluripotent stem cell (iPSC)-based treatments

**DOI:** 10.1186/s13287-024-04065-9

**Published:** 2024-11-21

**Authors:** Seohyun Jennie Song, Yoojun Nam, Yeri Alice Rim, Ji Hyeon Ju, Yeowon Sohn

**Affiliations:** 1 Seoul National University, 1 Gwanak-Ro, Gwanak-Gu, Seoul, 08826 Republic of Korea; 2 CiSTEM Laboratory, Convergent Research Consortium for Immunologic Disease, Seoul St. Mary’s Hospital, College of Medicine, The Catholic University of Korea, Seoul, 06591 Republic of Korea; 3 YiPSCELL Inc., L2 Omnibus Park, Banpo-Dearo 222, Seocho-Gu, Seoul, 06591 Republic of Korea; 4 Division of Rheumatology, Department of Internal Medicine, Seoul St. Mary’s Hospital, Institute of Medical Science, College of Medicine, The Catholic University of Korea, #505, Banpo-Dong, Seocho-Gu, Seoul, 06591 Republic of Korea; 5 Department of Biohealth Regulatory Science, Sungkyunkwan University, Suwon, South Korea

**Keywords:** Induced pluripotent stem cells, iPSC, Embryonic stem cells, Regenerative medicine, Clinical trials, Regulatory frameworks, Stem cell therapies, Advanced medicinal products

## Abstract

Stem cell therapies have emerged as a promising approach in regenerative medicine, demonstrating potential in personalized medicine, disease modeling, and drug discovery. Therapies based on induced pluripotent stem cells (iPSCs) particularly stand out for their ability to differentiate into various cell types while avoiding ethical concerns. However, the development and application of these therapies are influenced by varying regulatory frameworks across countries. This study provides a comparative analysis of regulations and research on stem cell therapies in key regions: The European Union (EU), Switzerland, South Korea, Japan, and the United States. First, the study reviews the regulatory frameworks on stem cell therapies. The EU and Switzerland maintain rigorous guidelines that prioritize safety and ethical considerations, which can hinder innovation. In contrast, the United States adopts a more flexible regulatory stance, facilitating the rapid development of stem cell therapies. South Korea and Japan take a balanced approach by incorporating practices from both regimes. These regulatory differences reflect each country’s unique priorities and impact the pace and scope of stem cell therapy development. Moreover, the study examines global trends in clinical trials on stem cell treatments based on data obtained from two sources: ClinicalTrials.gov and ICTRP. Findings indicate a significant growth in the number of clinical trials since 2008, particularly in that involving iPSCs. Therapeutic studies involving iPSCs predominantly target conditions affecting the cardiovascular and nervous systems which are considered vital. The results put emphasis on the safety of stem cell treatments. Meanwhile, the number of such trials also varies by country. The United States and Japan, where relatively flexible guidelines on stem cell research are adopted, are in a leading position. However, countries in the EU fall behind with rigorous regulations imposed. This reflects the need for more flexible regulatory guidance for active development of stem cell therapies. The findings underscore the importance of legal frameworks in facilitating innovation while ensuring safety. Regulatory agencies in different countries should collaborate to achieve a balanced global standard to ensure the safe and efficient advancement of stem cell therapies. Global regulatory convergence will promote international collaboration in research and the applicability of new treatments.

## Background

Stem cells are characterized by their ability to undergo self-renewal and differentiate into a multitude of cell lineages [1, 2]. Recently, therapies based on stem cells have demonstrated potential in treating chronic diseases as well as severe tissue impairments [3]. Owing to their plasticity, unlimited potential for replication, and ease of genetic modification, stem cell therapies have been revolutionary in managing human illnesses previously considered difficult to treat with conventional methods [4].

In general, stem cells are classified into three major categories: adult stem cells, embryonic stem cells (ESCs), and induced pluripotent stem cells (iPSCs). Adult stem cells are multipotent and can differentiate into a limited range of specific cell types. In contrast, ESCs and iPSCs are pluripotent, exhibiting the capacity to differentiate into any cell or tissue type.

ESCs have been a subject of considerable interest since the first discovery of stem cells in the 1960s. The study of ESCs has provided a deeper understanding of the processes governing cell reproduction and differentiation. Especially, ESC treatment has been promising in clinical trials [5]. Recent clinical trials involving ESCs have exhibited positive outcomes in treating various dysfunctions or injuries in different organs by promoting tissue regeneration, such as in endothelial dysfunction and spinal cord injury [6, 7].

However, the application of ESCs in research and clinical settings is limited by ethical considerations and technological challenges [8]. Induced pluripotent stem cells (iPSCs) generated from reprogrammed somatic cells could serve as a viable alternative to ESCs, as they share similar characteristics with ESCs but do not raise ethical issues or immunological rejection concerns associated with ESCs [9, 10]. iPSCs have also proven to be one of the most useful cells in the field of regenerative medicine, particularly in the area of personalized medicinal cell therapies [11, 12].

The technology behind iPSCs is rapidly advancing with its applications expanding to focus on disease modeling, drug discovery, and cell therapy development [13, 14]. Researchers are studying the maturation, aging, and metabolism of iPSC-derived cells to better understand the pathological features and mechanisms observed in patients [15]. For example, iPSCs have offered a unique model for investigating tumorigenesis and cancer treatments, as they can be generated from adult cells containing specific cancer-related mutations and be used to create disease-specific cell lines [16]. Of course, iPSC technology also come with certain limitations. To illustrate, the variability between cell lines derived from the same individual as well as the potential for genetic and epigenetic anomalies pose significant challenges in obtaining reliable results, particularly in investigating complex disorders like psychiatric illnesses [17]. However, iPSCs still remain among the most promising types of cells in stem cell therapies.

Significant advances in stem cell research have enabled a large amount of clinical trials involving human iPSCs in several countries, including the United States, South Korea, and Japan.

Meanwhile, clinical research and market placement of stem cell therapies, including those involving iPSCs, require the approval of competent local authorities [18, 19]. Consequently, it is essential to understand pertinent regulations in each country to facilitate the research and development of iPSC-based treatments. This entails an examination of legislations pertaining to stem cell-based advanced medicinal products as well as an investigation of the trend in the development and market placement of such products through therapeutic studies on iPSC-based treatments.

## Rules and regulations on stem cell therapies

### Regulatory frameworks around stem cell therapies

**Fig. 1:**
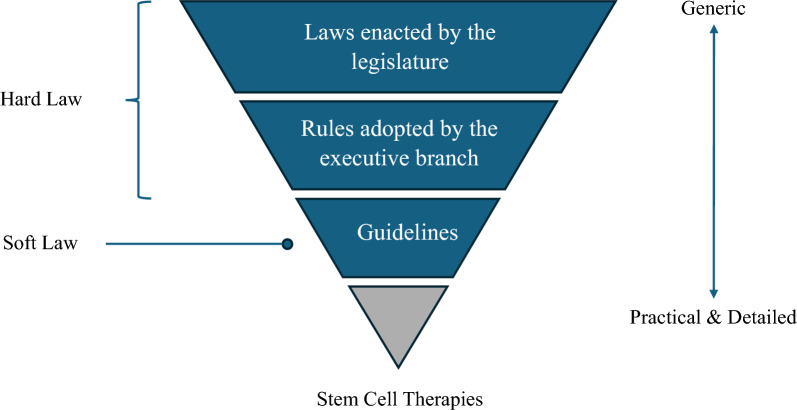
Regulatory frameworks around stem cell therapies. The figure illustrates the three regulatory layers on stem cell therapies, with the most superior and precedential at the top of the triangle. The laws enacted by the legislature are the most generic and legally binding, whereas guidelines are the most practical and autonomous.

The regulatory framework for stem cell therapy is structured in three tiers. The following represents the three layers ordered from most to least superior and precedential (Fig. 1). The first category consists of legislation enacted by the legislature, such as the parliament or congress. The second layer comprises regulations adopted by the executive branch, which are consistent with the laws set forth by the legislature. The final layer consists of guidelines and guidance notes published by regulatory entities. This final layer is regarded as "soft law", which is technically not legally binding but is expected to be adhered to in practice during the research, development, manufacturing, and clinical trial phases of stem cell therapies. In contrast, the first two are regarded as "hard law", and noncompliance with such regulations may result in penalties under national law.

The first regulatory layer, comprising laws enacted by the legislature, is designed to provide a generic framework for regulating stem cell therapies. The legislation establishes general guidelines for advanced regenerative products or cell-based therapies; however, it does not specifically address stem cell therapies. The most practical and granular guidance on the research and development of stem cell treatments, including those based on iPSCs, is defined in the final layer. A comprehensive list of the most recent guidelines published by the relevant authorities in each country is provided in Table 1.

**Table 1:** Guidelines on stem cell therapies

Country	Guidelines
EU	1. Reflection paper on stem cell-based medicinal products (2009 [84]) 2. Guideline on the minimum quality and non-clinical data for certification of advanced therapy medicinal products (2010 [85]) 3. Guideline on quality, non-clinical and clinical requirements for investigational advanced therapy medicinal products in clinical trials (2024 [86])
Switzerland	Opinion 06/2002: Research on embryonic stem cells (2002 [87])
South Korea	1. Guideline in Quality, Non-clinical and Clinical Assessment of Stem Cell Therapy Product (2014 [88]) 2. Considerations in Tumorigenicity Assessment of Stem Cell Therapy Product (2022 [89]) 3. Guideline on The Requirements for Quality Dossier of Cell and Gene Therapy Products (2022 [90])
Japan	1. Guidelines on clinical research using human stem cells, MHLW Notification No. 425 (2006 [91]) 2. The technical guidance for quality, non-clinical and clinical studies of regenerative medical products (human cell-processed products), PSEHB/MDED Administrative Notice No. 0614043 (2016 [91]) 3. Points for certified special committees for regenerative medicine to consider when evaluating tumorigenicity assessment in provision plans of regenerative medicine using human pluripotent stem cells (2021 [92])
United States	1. Regulatory considerations for human cells, tissues, and cellular and tissue-based products: Minimal manipulation and homologous use: Guidance for industry and Food and Drug Administration staff (2020 [93]) 2. Potency assurance for cellular and gene therapy products: Draft guidance for industry (2023 [94]) 3. Considerations for the use of human- and animal-derived materials in the manufacture of cellular and gene therapy and tissue-engineered medical products: Draft guidance for industry (2024 [95])

Albeit being practical and specific, the guidelines on stem cell therapy in various countries are not much different in scope and substance. Most guidelines address the following points: safety and efficacy requirements of raw materials throughout the development process, quality control measures during manufacturing, and potential considerations during non-clinical and clinical trials. In most countries, guidance on these matters is provided in a general and conservative manner, with a focus on ensuring the safety of stem cell therapy [20]. It is therefore evident that the role of competent authorities in interpreting these guidelines, as well as in adjudicating and approving products case by case, is imperative. Nonetheless, it is the legislative regulations and acts that delineate the authorities responsible for monitoring, engaging in, and granting approval in each step of the developmental process of stem cell-based products [19]. Also, they stipulate specific timelines and procedures that the competent authorities should adhere to during that process [21]. In this regard, the first layer is of particular importance when comparing the regulatory frameworks for stem cell therapies across countries.

**Fig. 2:**
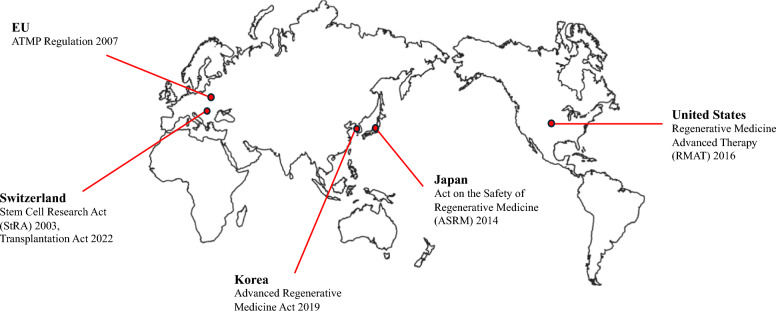
Legislative regulations on stem cell therapies. The figure shows the most recent legislative regulations on stem cell therapies in each of the five regions: the United States, Japan, Korea, Switzerland, and the EU.

Especially, the following regions are of particular interest in the comparative analysis of legislative regulations, as they are leading in the field of stem cell research and technology [22]: the European Union (EU), Switzerland, Korea, Japan, and the United States. Each country’s regulatory policies on stem cell-based products reflect its unique cultural values [23] in balancing ethical and safety concerns with scientific progress. The EU puts greater emphasis on ethical concerns, especially around the use of human embryos in research. For example, the European Biopatent Directive (Directive 98/44/EC on the Legal Protection of Biotechnological Inventions) prohibits patents on inventions involving the use of human embryos for commercial purposes [24]. Similarly, the Dickey-Wicker Amendment in the United States prohibits federal funding for research that involves the creation or destruction of embryos. However, California’s Proposition 71 focuses more on scientific progress, allocating a significant amount of state funding to support embryonic stem cell research [25]. Figure 2 presents the most recent legislative regulations on stem cell therapies at the national level across the five countries.

Although all listed countries are sovereign states with the authority to govern themselves, member states of the EU (e.g., France and Germany) are required to incorporate EU directives into their national legislation [26]. While these countries retain their sovereignty, they have granted the EU the authority to enact legislation binding on member states, including regulations on stem cell research and medicinal products [27]. In the following sections, any reference to the EU encompasses regulations enacted at the EU level and thus applied to all member states.

Some countries are subject to international agreements, such as human rights treaties, in regulating the development and the use of regenerative medicine including stem cell-based products. The 1997 Biomedicine Convention (Oviedo Convention), for instance, is a human rights regime that governs practices related to biomedicine and was implemented within the framework of the Council of Europe (CoE) [28]. This regime includes provisions pertaining to iPSC-based therapies that must be adhered to by ratified member states [29]. An intriguing aspect of international law is that these conventions are self-binding, which implies that even countries within the EU have the option to decline the ratification of such regimes. To be specific, France and Switzerland ratified the Oviedo Convention, whereas Germany did not [30]. Meanwhile, this is the first and the only international convention in bioethics [31]; therefore, countries outside the EU are not bound by international laws or treaties pertaining to iPSC-based therapies.

### Comparative analysis of regulations on stem cell-based medicinal products

The regulatory frameworks governing the manufacturing, clinical trial, and market authorization of stem cell-based medicinal products vary considerably across different countries. Overall, the EU and Switzerland have more rigorous regulations pertaining to stem cell-based medicinal products [32]. First, a manufacturing license is a prerequisite for initiating the manufacturing process for both clinical trials and market placements. Second, clinical trials are approved by using a prior authorization model [33]. Third, the modification of germline cells using stem cell therapy is prohibited by law in several states; France and Switzerland ratified the Oviedo Convention; and Germany enacted the Embryo Protection Act [34, 35].

On the other hand, the United States is more progressive and open to the research and development of stem cell therapies, including those involving iPSCs. First, a manufacturing license is not required for neither investigational nor marketable products. Second, a prior notification model is employed for clinical trials of advanced medicinal products and Accelerated Approval is permitted for such therapies before market placement [36, 37]. Third, the legislative body does not ban germline cell modification by law; rather, the responsibility for regulating this practice falls on the guidance level of the Food and Drug Administration (FDA) [38]. Overall, regulatory science is well-established in the United States. Therefore, instead of imposing researchers a rigorous and unilateral scientific standard by the legislation, the FDA examines each cell-based therapy case by case with more detailed but more flexible standards for its approval. The objective of regulators in the United States is to achieve a balance between safety control and scientific progress [19].

Both South Korea and Japan have implemented regulations that reflect the spirit of both the United States and the EU, as laws and executive guidance often reference precedents set by other countries and attempt to follow global standards. Nevertheless, South Korea is more like the EU in that it places a strong emphasis on ensuring safety, whereas Japan enforces relatively lenient laws just as the United States. To be specific, South Korea adopts a prior authorization model for clinical trial approval and, in accordance with the Bioethics and Safety Act (Bioethics Act), approves clinical trials of advanced cell-based medicinal products only if they are intended to cure a hereditary disease or if no alternative exists to the disease in question [39]. Also, germline therapies are strictly prohibited in South Korea. Conversely, Japan follows a prior notification model and MLHW guidelines allow germline editing for research purpose [40].

**Fig. 3:**
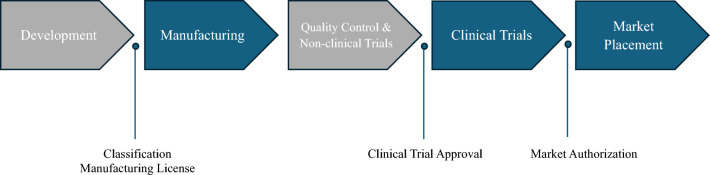
Developmental process of stem cell-based medicinal products and related regulatory procedures. The figure displays the six steps in the development process of stem cell therapies: development, manufacturing, quality control, non-clinical and clinical trials, and market placement. Also, the steps colored in blue have prerequisites, which are denoted in the bottom; for example, classification and manufacturing license are prerequisites for manufacturing.

The development of stem cell treatments consists of five phases, as illustrated in Fig. 3. Legal and regulatory frameworks define the requirements for each stage of the development process. The manufacture, clinical trials, and market placement require the acquisition of a manufacturing license, approval of a clinical trial, and market authorization, respectively. The following section provides a comparative analysis of these aspects across different countries; the results are summarized in Table 2.

**Table 2:** Regulations on each step of developmental process

	EU	Switzerland	South Korea	Japan	United States
Classification	Categorization				
	Advanced Therapy Medicinal Product (ATMP)		Advanced Regenerative Medicine	Regenerative Medical Products	Human cells, tissues, and cellular and tissue-based product (HCT/P)
	Procedure				
	Required to apply for classification with the EMA		N/A	N/A	Required to register with the FDA
Manufacturing License	Required before manufacturing investigational and marketable products		Required before manufacturing marketable products Not Required for investigational products	Required before manufacturing investigational and marketable products	Not Required; but must submit "Chemistry, Manufacturing, and Control (CMC) " information
Clinical Trial Approval	Prior Authorization			Prior Notification	
Market Authorization	Special mentoring				
	PRIME program	N/A	N/A	N/A	Fast Track program
	Priority review				
	150 Days	N/A	60 days	N/A	6 months
	Provisional authorization				
	Conditional Marketing Authorization (CMA)	Temporary Authorization	Conditional Approval	Conditional and Time-limited Approval	Accelerated Approval
	Clinical trials not required			Phase II Clinical Trials Required	Clinical trials not required

#### Classification

The term "classification" is used to describe the process of categorizing cell-based treatments into the category of stem cell treatments. The advent of new technologies has obscured the distinction between stem cells and other cell types, with an expanding range of cells now being identified as stem cells [41]. Consequently, it is not always evident whether a particular cell-based treatment can be classified as stem cell treatment. Nevertheless, classification represents a pivotal initial stage in the developmental process, as a treatment must be designated as a stem cell treatment in order to be subject to regulations pertaining to stem cell-based medicinal products in subsequent phases, including the acquisition of a manufacturing license or the approval of non-clinical and clinical trials [42]. The following section provides a detailed explanation of the classification process for stem cell-based products across different countries.

In the United States, stem cell therapies, including those based on iPSCs, are classified as human cells, tissues, and cellular and tissue-based products (HCT/Ps), as they involve the alteration of human cells to treat diseases [43, 44]. There are two categories of HCT/Ps: 351 HCT/Ps and 361 HCT/Ps. The former is subject to regulation under section 351 of the Public Health Service Act (1994) [43, 45], whereas the latter is regulated under section 361. This distinction hinges on whether the manufacturing process entails merely a "minimal manipulation" of the cell or tissue. A product is classified as 361 HCT/P if it is manufactured using minimal manipulation and is intended for homologous use [43, 46]; otherwise, the product is classified as 351 HCT/P. Stem cell therapies, including those based on iPSCs, are classified as 351 HCT/Ps because they require "more than minimal manipulation" of human cells [47, 48], and thus do not qualify as 361 HCT/Ps. The FDA requires the entities engaged in the development of 351 HCT/Ps to register the treatment for classification before manufacturing the product [45, 49].

In the EU, stem cell treatments are designated as advanced therapeutic medicinal products (ATMPs) [42, 50]. ATMPs are classified into three categories: (a) gene therapy, (b) somatic-cell therapy, and (c) tissue-engineered medicines [42, 51]. Products derived from iPSCs are classified as tissue-engineered products due to the substantial modification of the cells' "biological characteristics, physiological functions, or structural properties" [52]. The European Medicine Agency (EMA)'s Committee for Advanced Therapies (CAT) is responsible for adjudicating the classification of medicinal products as ATMPs. This is done within 60 days after the company in charge of developing the treatment applies for consultation. CAT publishes the classification results as reports to let other applicants reference these reports in the future [53–55].

In Japan, stem cell-based medicinal products are classified as "regenerative medical products [47, 56]", which encompass both gene therapy and cell-processed products intended for commercialization. The Ministry of Health, Labor, and Welfare (MHLW) is vested with the authority to decide the classification of treatments as regenerative medical products, as delineated in the Pharmaceuticals and Medical Devices Act (2013) [56]. A noteworthy distinction is that if a stem cell therapy is not intended for commercialization but is instead used to treat specific patients at the discretion of the physician, it is classified as "specified processed cells" in accordance with the provisions of the Regenerative Medicine Safety Act (2013) [57].

In South Korea, stem cell treatments are classified as advanced regenerative medicine in accordance with the Advanced Regenerative Medicine Act (2019) [58, 59]. Advanced regenerative medicine is defined as "cell therapy, gene therapy, or tissue engineering therapy" that is used to "regenerate, restore, or establish a person's physical structure or function, or to treat or prevent diseases" [58]. In accordance with this legislation, the "Advanced Regenerative Medicine and Advanced Biological Products Policy Review Committee" has the authority to classify stem cell therapies as advanced regenerative medicines [58].

#### Manufacturing license

Regulations pertaining to the manufacture of stem cell therapy products can be classified into two principal categories: the production of investigational products for clinical trials and the production of medicinal products intended for commercial distribution.

First, except for the United States and South Korea, every country requires a license to manufacture investigational products for clinical trials. Obtaining a manufacturing license is a prerequisite for initiating the clinical trial phase of stem cell therapy development. In the United States, manufacturing licenses are not a prerequisite for conducting clinical trials. Instead, investigators must submit information pertaining to the "Chemistry, Manufacturing, and Control" of the products in question to the FDA [60]. South Korea also provides an exception to the manufacturing license requirement for investigational advanced regenerative medicines, including stem cell-based therapeutic products [61]. In most countries, the same authority is responsible for both the review of clinical trial applications and the granting of manufacturing licenses. Japan provides a case in point, with the MLHW assuming oversight of both functions. In Germany, however, the process of obtaining a manufacturing license requires the applicant to receive approval from two authorities: the regional competent authority within the state and the Paul-Ehrlich Institute (PEI). The regional authority has jurisdiction over all medicinal products, whereas the PEI has authority over products "manufactured using genetic engineering" [62].

Second, a distinct manufacturing license is required to produce iPSC-based medicinal products intended for commercialization in countries other than the United States [63]. Most countries have separate Good Manufacturing Practice (GMP) guidelines for stem cell-based medicinal products. These guidelines present an additional set of requirements to the manufacturers on top of the requirements for generic pharmaceutical products. For example, the EU has published a document titled "Good Manufacturing Practice for Advanced Therapy Medicinal Products," which provides detailed specifications regarding the manufacture of cell and gene therapy products for market placement. In the United States, no manufacturing license is required; however, the FDA has published manufacturing guidance for cell-based therapy products.

#### Clinical trial approval

Approval processes for clinical trials of stem cell-based products can be classified into two models: prior authorization and prior notification. Most countries adhere to the "prior authorization" model, in which researchers must obtain approval from the relevant authority before the commencement of clinical trials [33]. The competent authority will assess the safety of the clinical trial to ensure that the methodology follows good clinical practices and does not pose any substantial risk to the participants. Also, it will review the ethical implications of the proposed treatment, specifically regarding editing the human genome. The prior authorization model provides a stronger guarantee that the relevant authority has comprehensively examined the procedures and substance of the trial before the initiation of the trial itself.

Meanwhile, the United States and Japan adhere to the "prior notification" model, wherein researchers are permitted to notify the pertinent authorities and subsequently commence the trial after a designated waiting period has elapsed [64, 65]. In contrast to the prior authorization model, the prior notification model does not require the approval of competent authorities, such as the MLHW (Japan) and FDA (United States), for the researcher to initiate the trial. However, the authorities may subsequently notify the researcher to suspend the trial if it is deemed unsafe or unethical upon review. In the United States, if the FDA decides and notifies the applicant of approval before the conclusion of the waiting period, a clinical trial may commence at an earlier date [66].

Given the advanced nature of stem cell therapies, particularly those involving iPSCs, and the potential for previously unidentified risks, competent authorities often provide consultation programs for clinical trials at the earliest stages of development. At various stages of the clinical trial, authorities may provide substantial advice on matters such as the manufacturing process of investigational therapy products and data privacy regulations. While the advice provided by competent authorities is not legally binding, researchers frequently seek consultation not only because it is advantageous to receive feedback at an early stage of the clinical trial, but also because it facilitates compliance with local regulations governing research. Unlike authorities in other countries, the U.S. FDA provides structured consultation programs, including pre-IND meetings and the INTERACT (Initial Targeted Engagement for Regulatory Advice on CBER Products) program [67, 68].

#### Market authorization

All countries have established a regulatory framework that requires a review process for authorization before stem cell therapy products can be placed on the market. Nevertheless, given that cell-based therapy is regarded as an advanced treatment distinct from other biopharmaceutical products, competent authorities have granted three exceptional procedures to accelerate the review process and facilitate the development.

First, competent authorities provide specialized mentoring and attention for the review of stem cell treatments, including iPSC-based medicinal products. This entails more frequent communication between the researcher and relevant authorities, wherein the researcher can receive detailed feedback and guidance. One example is the EMA's PRIME (Priority Medicines) program, which provides comprehensive support throughout the approval process [69]. Another example is the FDA's Fast Track program, which offers professional guidance to investigators from the outset of the clinical trial phase to the approval stage for market placement.

Second, stem cell-based therapeutic products may benefit from accelerated approval. The time required for approval is significantly reduced if the product undergoes an accelerated procedure. South Korea recently enacted an accelerated review process in the Advanced Regenerative Medicine Act (2019), which requires the Ministry of Food and Drug Safety to adjudicate applications for the market authorization of advanced regenerative medicines within 60 days of submission [70]. In addition, the FDA offers the Priority Review program, which reduces the timeline for judging a Biological License Application (BLA) from 10 to 6 months for advanced medicinal products [71].

Third, a provisional authorization policy is offered by competent authorities, whereby the applicant is partially relieved of the burden of providing evidence related to the efficacy and safety of the medicinal product. To illustrate, the FDA's Accelerated Approval program permits BLA applicants to demonstrate the efficacy of only intermediate and surrogate endpoints, instead of conventional endpoints [37]. Policies pertaining to provisional authorization vary considerably across countries. To be specific, Japan mandates the completion of phase 2 clinical trials, whereas other countries, including the United States and the EU, require no clinical trials for provisional authorization [72]. The applicant must demonstrate the efficacy and safety of the medicinal product in clinical trials within a certain period of time after the provisional authorization is granted; the duration of this buffer period varies by country. Japan has the longest buffer period, as proof is required within 7 years of authorization [72]; the EU requires proof within one year, with the possibility of renewal. The exceptional approval procedures for iPSC-based therapeutic products permit testing of investigational iPSC-based treatment methods in clinical trials. Authorities have implemented a minimal set of safeguards to ensure the safety, efficacy, and quality of cell-based medicinal products, upholding the validity of scientific research and protecting patient welfare.

#### Regulations on iPSC-based germline treatment

iPSCs can be genetically modified and subsequently employed to derive gametes, facilitating the production of genetically modified germline cells [73]. Although technically feasible, the prevailing view among the scientific community is that germline treatment is ethically unacceptable and condemnable. Consequently, most countries in this analysis have enacted legislation or adopted guidance that prohibits the use of iPSCs in germline cell therapies. For example, South Korea enacted the Bioethics Act, which explicitly prohibits germline therapies and stipulates that any individual engaging in such activities may be subject to criminal prosecution. Germany also enacted the Embryo Protection Act, which unequivocally prohibits modifications of germline cells [34, 39]. Some countries in the EU as well as Switzerland also adopts Oviedo Convention which bans germline therapy.

Meanwhile, in Japan, regulations on germline cell therapy are comparatively lenient yet remain subject to guidelines published by the MLHW [40]. These guidelines permit germline editing for research aimed at treating genetic diseases but impose restrictions on reproductive applications and clinical testing [74]. In the United States, although there are no formal laws or regulations that restrict germline cell therapy, the FDA prohibits the use of advanced therapies for editing germline cells [38]. The regulatory frameworks governing germline cell treatment in different countries are presented in Table 3.

**Table 3:** Regulations on germline cell therapy

Rigorous ← Level of Control → Lenient				
EU	Switzerland	South Korea	Japan	United States
CoE’s biomedicine convention (Oviedo convention)		Bioethics and Safety Act strictly prohibits germline therapies	MLHW guidelines allows germline editing only for research purposes	No law or regulation; FDA prohibits approving and funding germline cell therapies
Embryo Protection Act (Germany)	Reproductive Medicine Act			

## Therapeutic studies involving clinical trials on iPSC-based treatments

### Global trends in clinical studies on stem cell therapies

#### Clinical studies involving stem cells by cell type

**Fig. 4:**
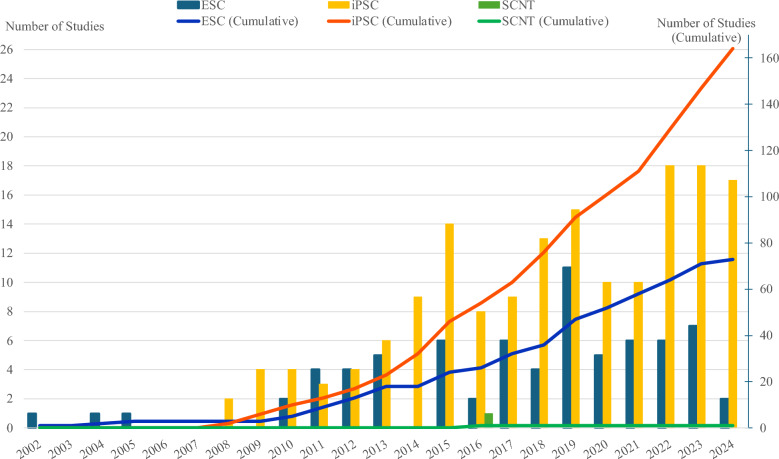
Global Trends in Clinical Studies Involving Different Types of hPSCs. The graph shows the trend in the number of clinical studies worldwide involving different types of human pluripotent stem cells (hPSCs), including ESCs, iPSCs, and SCNTs. Data were collected from two sources: ClinicalTrials.gov and ICTRP. The bar chart indicates the number of clinical trials for each hPSC type by year, whereas the line graph shows the cumulative number of clinical trials since 2002.

To gain insight into the global landscape of stem cell therapies, it is essential to examine data on clinical studies using stem cells. Figure 4 illustrates the temporal trend in the number of clinical studies worldwide involving different types of human pluripotent stem cells (hPSCs) over the past two decades. Data on clinical trials were collected on June 20th, 2024 from two sources: ClinicalTrials.gov, managed by the US National Library of Medicine, and the International Clinical Trials Registry Platform (ICTRP), maintained by the World Health Organization (WHO).

Three types of hPSCs were subject to investigation: ESCs, iPSCs, and SCNTs. A search for the keywords "embryonic stem cells," "induced pluripotent stem cells OR iPSC," and "somatic cell nuclear transfer OR SCNT" yielded 73, 164, and 1 results, respectively, after removing duplicates between ClinicalTrials.gov and ICTRP. The keyword “ESC” was excluded when searching for studies involving ESCs because “ESC” is widely used in other contexts and therefore yields a lot of irrelevant results. The results included both the observational and interventional studies. Subsequently, the studies were aggregated by year based on the date of enrollment. This enabled an investigation of the evolution of stem cell types that garnered the most attention from the research community over time.

As illustrated in Fig. 4, the utilization of hPSCs in clinical trials emerged in the early 2000s. Since 2008, the number of relevant studies have demonstrated a consistent upward trajectory, particularly those involving iPSCs and ESCs. More recently, clinical studies based on iPSCs constitute the vast majority, demonstrating a persistent upward trend. For the past 10 years, iPSCs accounted for more than 60% of all clinical studies involving hPSCs; the ratio even recorded up to 100% in 2014 and 89% in 2024. The recent exponential growth in the number of clinical trials involving iPSCs indicates a heightened focus on iPSCs within the field of stem cell research. Since their discovery, iPSCs have been considered a groundbreaking technology with the potential to treat several incurable diseases, including malignant tumors and rare genetic disorders [75, 76].

The generation of iPSCs involves the injection of reprogramming factors into adult somatic cells, which are then converted into an embryonic-like state [77]. Owing to their capacity for regeneration, iPSCs can differentiate into any type of cell, providing an inexhaustible source of individualized tissue and organ replacement therapy [78]. Furthermore, iPSCs can be used as powerful tools for the development of personalized medicines that target genetic diseases [79]. By modeling diseases using cell lines derived from patients with genetic mutations, researchers can ascertain the underlying mechanisms of the disease and develop treatments that address specific genetic defects in each patient [80]. For example, patient-derived iPSCs and organoids are recently preferred as an effective source to figure out the accurate mechanisms of inherited retinal diseases (IRDs) and are considered to have significant therapeutic implications [81]. Moreover, iPSCs are highly effective for drug screening because they enable researchers to assess the safety and efficacy of potential treatments in a more realistic setting than conventional techniques, such as those based on animal models; researchers may use tissues and organs derived from patients’ iPSCs [16]. In this sense, iPSC-based therapies are expected to play a pivotal role in the future advancement of stem cell research.

#### Clinical studies on stem cell therapies by country

In order to provide a deeper insight into how active stem cell research is in different countries, an investigation of the trend in clinical studies involving stem cell therapies was conducted by country. This analysis effectively contextualizes the iPSC-specific trend analysis in the later section, as it enables the comparison of the number of clinical trials involving iPSCs to that of clinical studies based on stem cells in each country. A methodology analogous to that previously outlined was employed to collate clinical research data from two principal sources: ClinicalTrials.gov and the ICTRP [82].

**Fig. 5:**
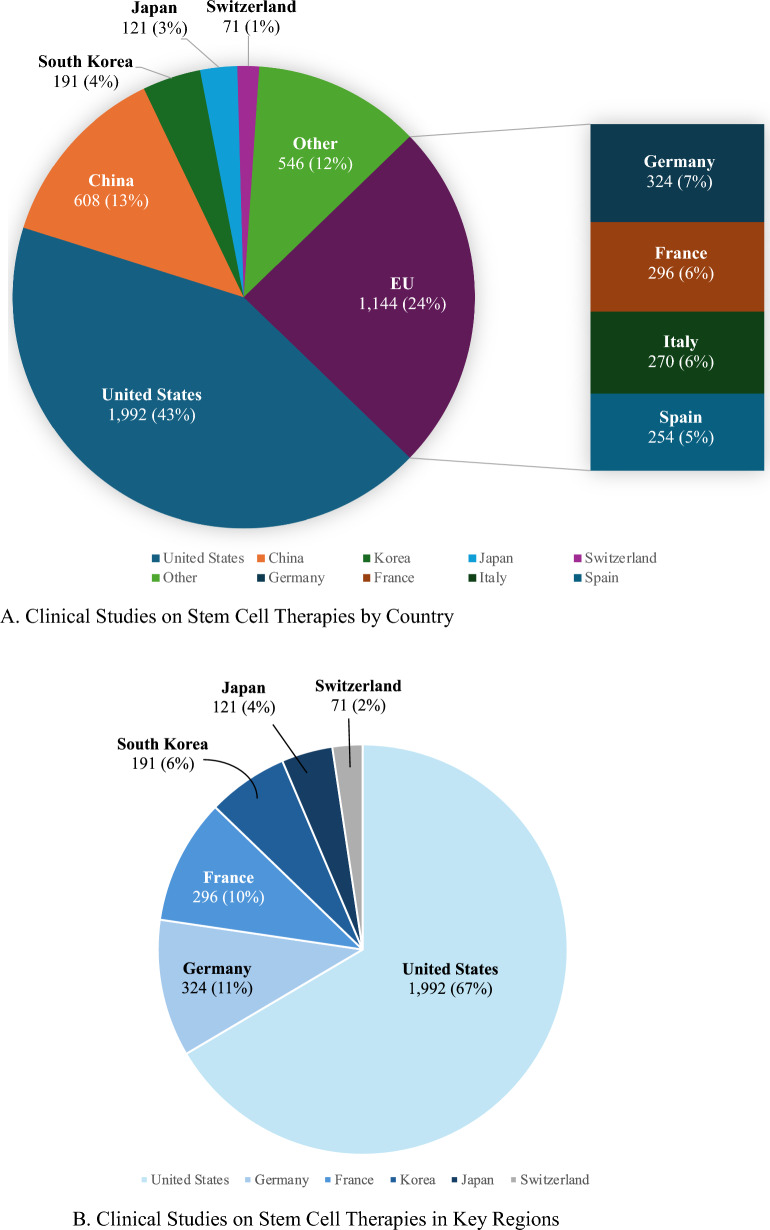
**A** Clinical studies on stem cell therapies by country. The figure shows the distribution of clinical trials on stem cell therapies across different countries. Data on clinical trials were collected from two sources: ClinicalTrials.gov and ICTRP. **B** Clinical studies on stem cell therapies in key regions. The figure shows the number and the relative proportion of clinical trials conducted in key regions denoted in this study, which include: the EU (Germany and France), Switzerland, South Korea, Japan, and the United States.

Data were collected on June 20th, 2024. A search for clinical studies was conducted using the keywords "stem cell therapies OR stem cell treatments," resulting in 3,329 and 1,938 results from ClinicalTrials.gov and ICTRP, respectively. Following the removal of duplicate studies identified by both sources, 4,673 results were retrieved. The studies were matched based on trial ID and study title to identify the duplicates. Figure 5 illustrates the country-specific distribution of the 4,673 clinical studies retrieved using this methodology. As illustrated in Fig. 5A, the United States led with 1,992(43%) studies; the EU followed by 1,144(24%) with Germany, France, Italy, and Spain having 324(7%), 296(6%), 270(6%), 254(5%) studies, respectively. China trailed behind with 608(13%). South Korea had 191(4%); Japan had 121(3%); and Switzerland had 71(1%). Figure 5B summarizes the results of only the five key regions addressed in this study: The United States, the EU (Germany and France), South Korea, Japan, and Switzerland.

### Global trends in clinical studies on iPSC-based treatments

#### Classification of clinical studies involving iPSCs

iPSCs are playing an essential role and have recently been the most popular cell types used in the field of stem cell research. Therefore, the number of clinical trials that involve iPSC-based treatments was used as the proxy for the global trend in the research and development of stem cell therapies. This section provides a comprehensive assessment of clinical trials involving iPSCs, with data collected on June 21st, 2024, from the same two sources as above: ClinicalTrials.gov and ICTRP. Subsequently, the studies were classified according to three different criteria.

**Fig. 6:**
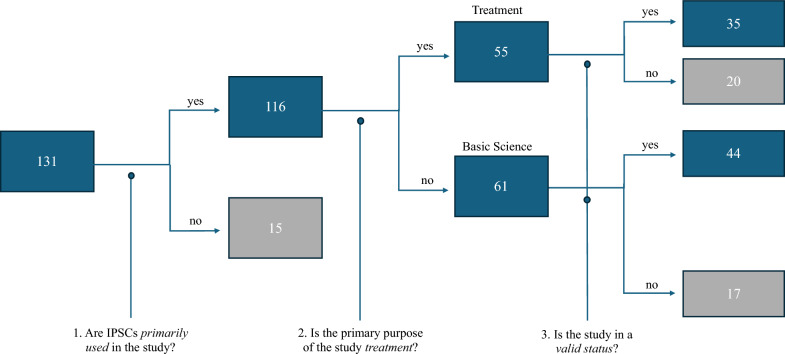
Classification tree of clinical studies involving iPSCs. The figure illustrates the classification of 131 clinical studies related to iPSCs, retrieved from ClinicalTrials.gov and ICTRP using a keyword search on "induced pluripotent stem cells OR iPSC." The flow chart shows three criteria used in the classification and the number of studies that fall into each category. The figure also shows the breakdown of 44 studies in which iPSCs were primarily used, treatment was not the primary purpose, and are considered to be in valid status; this sheds light on the objectives of basic science studies in which iPSCs were used.

A search for the keyword "induced pluripotent stem cells OR iPSC" yielded 131 studies identified as clinical trials involving iPSCs. Specifically, 113 studies were sourced from ClinicalTrials.gov, whereas 36 were sourced from ICTRP. In ClinicalTrials.gov, the keyword was entered to the filter "Intervention/treatment," as we intended to focus only on therapeutic studies involving certain treatments. In ICTRP, the “Phases” filter was used to only search for clinical studies reported to be in phases 0 to 4. Among these, 18 duplicate studies were removed. Figure 6 illustrates the subsequent analysis of the 131 studies that were classified according to three standards. During this process, studies that did not meet the standards were excluded to only identify valid studies using iPSCs for therapeutic applications.

The first criteria for classification were the *primary utilization* of iPSCs. Specifically, we examined whether iPSCs were (a) identified as essential and critical materials in the study and (b) emphasized in the primary outcome measure. 15 studies that did not meet the criteria were excluded upon further analysis. These studies include those in which iPSCs were not used, were used only for somatic cell or blood sample collection, or were not highlighted in the primary outcome of the study.

The second criterion for classification was whether the study focused primarily on *treatment purposes*. A study was deemed to have a treatment purpose if it was (a) conducted to assess the safety, efficacy, and side effects of iPSC-based therapies and (b) interventional in that it involves the direct administration of iPSCs into the subject's body. Most of these studies were labeled as "Primary Purpose: TREATMENT" in the "Study Design" column. In accordance with the aforementioned criteria, 61 studies on basic science research were excluded. Examples of such studies include those focused on generating iPSCs, reprogramming or differentiating iPSCs into other cell lines, modeling target diseases, and creating cell banks.

The final standard pertains to whether the study is in a *valid status*. A study was deemed valid had it been completed (including those listed as "not recruiting" or "no longer recruiting" with the results available) or was still ongoing (those listed as "recruiting", "enrolling by invitation", and "actively not recruiting"). The study was deemed invalid if the recruitment status was identified as one of the following: "terminated", "suspended", "pending", "withdrawn”, "not yet recruiting", or "unknown". Twenty out of the 55 treatment studies and 17 out of the 61 basic science studies were excluded based on the final classification criterion. The remaining 44 basic science clinical studies that satisfied this criterion consist of the following topics: 11 for generating iPSCs only for various future uses, 13 for reprogramming iPSCs, 12 for disease modeling, four for establishing cell banks, and four for other purposes.

A total of 35 studies met all three criteria, indicating that iPSCs were primarily used, the objective of the study was treatment, and the study itself was deemed valid. For simplicity, these studies are referred to as "iPSC treatment studies." A comprehensive list of iPSC treatment studies is provided in Table 4.

**Table 4:** Clinical trials involving iPSCs-based treatments

No.	Trial number	Study title	Phase	System	Condition	Enrollment	Start date	Sponsor	Country
1	NCT02923375	A Study of CYP-001 for the Treatment of Steroid-Resistant Acute Graft Versus Host Disease	1	Immune	SR-aGVHD	16	2017-03-01	Cynata Therapeutics Limited	Australia
2	NCT03841110	FT500 as Monotherapy and in Combination With Immune Checkpoint Inhibitors in Subjects With Advanced Solid Tumors	1	-	Solid Tumor	37	2019-02-15	Fate Therapeutics	United States
3	NCT04339764	Autologous Transplantation of Induced Pluripotent Stem Cell-Derived Retinal Pigment Epithelium for Geographic Atrophy Associated With Age-Related Macular Degeneration	1–2	Visual	Macular Degeneration	20	2020-09-23	National Eye Institute (NEI)	United States
4	NCT04363346	Study of FT516 for the Treatment of COVID-19 in Hospitalized Patients With Hypoxia	1	Respiratory	COVID-19	5	2020-05-14	Masonic Cancer Center, University of Minnesota	United States
5	NCT04396899	Safety and Efficacy of Induced Pluripotent Stem Cell-derived Engineered Human Myocardium as Biological Ventricular Assist Tissue in Terminal Heart Failure	1–2	Cardiovascular	Heart Failure	53	2020-02-03	University Medical Center Goettingen	Germany
6	NCT04537351	The Mesenchymal coviD-19 Trial: MSCs in Adults With Respiratory Failure Due to COVID-19 or Another Underlying Cause	1	Respiratory	COVID-19	14	2020-08-24	Cynata Therapeutics Limited	Australia
7	NCT04630769	FT516 and IL2 With Enoblituzumab for Ovarian Cancer	1	Reproductive	Gynecologic Cancer	3	2021-04-02	Masonic Cancer Center, University of Minnesota	United States
8	NCT04744532	iPSC-based Drug Repurposing for ALS Medicine (iDReAM) Study	1–2	Nervous	Amyotrophic Lateral Sclerosis	49	2019-03-19	Kyoto University	Japan
9	NCT04945018	A Study of iPS Cell-derived Cardiomyocyte Spheroids (HS-001) in Patients With Heart Failure (LAPiS Study)	1–2	Cardiovascular	Ischemic Heart Disease	10	2022-04-19	Heartseed Inc	Japan
10	NCT04982081	Treating Congestive HF With hiPSC-CMs Through Endocardial Injection	1	Cardiovascular	Heart Failure	20	2021-09-21	Help Therapeutics	China
11	NCT05182073	FT576 in Subjects With Multiple Myeloma	1	Hematologic	Myeloma	168	2021-11-24	Fate Therapeutics	United States
12	NCT05336409	A Study of CNTY-101 in Participants With CD19-Positive B-Cell Malignancies	1	Lymphatic	Non-Hodgkin Lymphoma	75	2023-01-24	Century Therapeutics, Inc	United States
13	NCT05445063	Safety and Efficacy of Autologous Transplantation of iPSC-RPE in the Treatment of Macular Degeneration	1	Visual	Macular Degeneration	10	2022-08	Beijing Tongren Hospital	China
14	NCT05566600	Allogeneic iPSC-derived Cardiomyocyte Therapy in Patients With Worsening Ischemic Heart Failure	1	Cardiovascular	Ischemic Heart Failure	32	2022-10-09	Help Therapeutics	China
15	NCT05643638	A Study of CYP-001 in Combination With Corticosteroids in Adults With High-risk aGvHD	2	Immune	aGVHD	60	2024-03-04	Cynata Therapeutics Limited	United States
16	NCT05647213	Autologous Induced Pluripotent Stem Cells of Cardiac Lineage for Congenital Heart Disease	1	Cardiovascular	Univentricular Heart Disease	50	2023-02-03	HeartWorks, Inc	United States
17	NCT05774509	Treatment of Non-ischemic Cardiomyopathies by Intravenous Extracellular Vesicles of Cardiovascular Progenitor Cells	1	Cardiovascular	HFrEF	12	2023-05-31	Assistance Publique—Hôpitaux de Paris	France
18	NCT05886205	Induced Pluripotent Stem Cell Derived Exosomes Nasal Drops for the Treatment of Refractory Focal Epilepsy	1	Nervous	Refractory Focal Epilepsy	34	2023-06-05	Peking Union Medical College Hospital	China
19	NCT05969717	Induced Pluripotent Stem Cell Derived Exosomes for the Treatment of Atopic Dermatitis	1	Integumentary, Immune	Atopic Dermatitis	20	2023-04-12	Peking Union Medical College Hospital	China
20	NCT06027853	Natural Killer(NK) Cell Therapy Targeting CLL1 in Acute Myeloid Leukemia	1	Hematologic	Myeloid Leukemia	24	2023-09-10	Zhejiang University	China
21	NCT06138210	The Effect of GD-iExo-003 in Acute Ischemic Stroke	1	Nervous	Ischemic Stroke	29	2024-05-27	Xuanwu Hospital, Beijing	China
22	NCT06299033	A Safety and Tolerability Study of Human Forebrain Neural Progenitor Cells Injection (hNPC01) in Subjects With Chronic Ischemic Stroke	1	Nervous	Ischemic Stroke	21	2023-11-09	Hopstem Biotechnology Inc	China
23	NCT06321198	A Trial to Evaluate the Safety and Preliminary Efficacy of iMesenchymal Stromal Cells(iMSC) in Subjects With SR-aGVHD	N/A	Immune	SR-aGVHD	12	2024-02-10	Anhui Provincial Hospital	China
24	NCT06344026	Phase 1/2a Study of ANPD001 in Parkinson Disease	1	Nervous	Parkinson's Disease	9	2024-01-23	Aspen Neuroscience	United States
25	NCT06359912	Safety and Preliminary Efficacy of Allogeneic Endothelial Progenitor Cells (EPCs) in Patients With Critical Limb Ischemia	1	Peripheral vascular	Critical Limb Ischemia	27	2024-04	Allife Medical Science and Technology Co., Ltd	China
26	NCT06367673	Natural Killer(NK) Cell Therapy Targeting CLL1 or CD33 in Acute Myeloid Leukemia	1	Hematologic	Myeloid Leukemia	24	2024-04-30	Zhejiang University	China
27	NCT06394232	Safety & Efficacy of Eyecyte-RPE™ in Patients With Geographic Atrophy Secondary to Dry Age-related Macular Degeneration	1–2	Visual	Macular Degeneration	54	2024-06-04	Eyestem Research Pvt. Ltd	India
28	ChiCTR2300072200	An exploratory clinical study of islet-like cell transplantation differentiated from autologous induced pluripotent stem cells for the treatment of type 1 diabetes	0	Endocrine	Type 1 Diabetes	Experimental group: 3	2023-06-11	Tianjin First Center Hospital	China
29	JPRN-jRCT2033210163	A phase I/II study of human induced Pluripotent Stem (iPS) cell-derived cardiomyocyte spheroids in patients with severe heart failure, secondary to ischemic heart disease, undergoing coronary artery bypass grafting	1–2	Cardiovascular	Ischemic Heart Disease	10	2021-06-23	Kaneko Takehiko	Japan
30	JPRN-jRCT2090220384	Kyoto Trial to Evaluate the Safety and Efficacy of iPSC-derived dopaminergic progenitors in the treatment of Parkinson's Disease	1–2	Nervous	Parkinson's Disease	7	2018-08-01	Kyoto University Hospital	Japan
31	JPRN-jRCT2091220385	Kyoto Trial to Evaluate the Safety and Efficacy of iPSC-derived dopaminergic progenitors in the treatment of Parkinson's Disease	3	Nervous	Parkinson's Disease	7	2018-08-01	Kyoto University Hospital	Japan
32	JPRN-jRCTa032200189	Safety study of induced pluripotent stem cell-derived cardiac spheres transplantation	0	Cardiovascular	HFrEF by Dilated Cardiomyopathy	3	2020-11-09	Shimizu Hideyuki	Japan
33	JPRN-jRCTa050190117	iPSC-derived platelet transfusion trial1	1	Hematologic	Aplastic Anemia	1	2019-05-14	Takaori-Kondo Akifumi	Japan
34	JPRN-jRCTa050210178	Clinical Research of allogeneic iPSC-RPE cell strip transplantation for RPE impaired disease	1–2	Visual	RPE impaired Disease	50	2022-11-21	Kurimoto Yasuo	Japan
35	JPRN-UMIN000033565	Kyoto Trial to Evaluate the Safety and Efficacy of Tacrolimus in the iPSC-based Therapy for Parkinson's Disease	3	Nervous	Parkinson's Disease	7	2018-08-01	Kyoto University Hospital	Japan

#### Comparative analysis of iPSC treatment studies by targeted disease

**Fig. 7:**
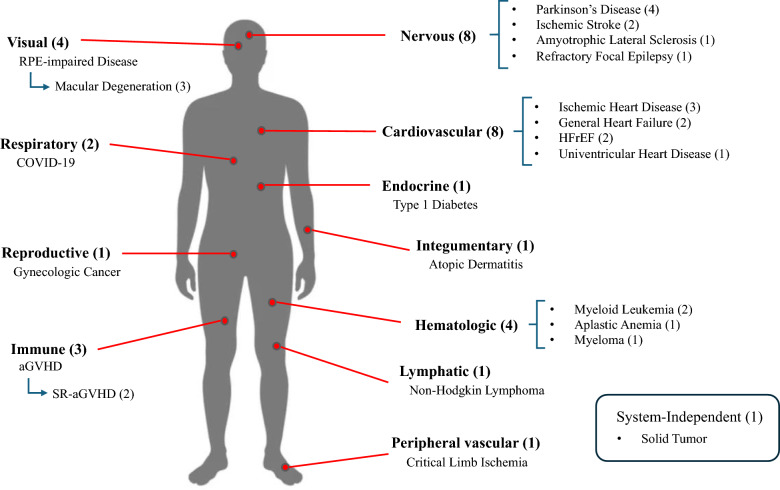
Number of iPSC treatment studies by relevant body system of targeted condition. The diagram shows the number of iPSC treatment studies targeting each condition and body system, such as the visual system or the nervous system.

To gain further insight into the trends observed in iPSC treatment studies, we first classified the studies according to the targeted condition(s). Figure 7 illustrates the number of studies aggregated by the body systems associated with the targeted diseases. Eight studies were identified as investigating treatments targeting the nervous system. Of these, four focused on Parkinson's disease {24, 30, 31, 35}, two on ischemic stroke {21, 22}, one on amyotrophic lateral sclerosis {8}, and one on refractory focal epilepsy {18}. (Numbers provided in braces correspond to assigned "No." of clinical studies targeting each condition in Table 4). Four studies focused on the visual system, with all four targeting retinal pigment epithelium (RPE)-impaired disease {3, 13, 27, 34}, three of which were related to macular degeneration {3, 13, 27}.

In addition, there were 14 studies on the circulatory system, encompassing the cardiovascular, hematological, lymphatic, and peripheral vascular systems. Eight studies focused on the cardiovascular system: three on ischemic heart disease {9, 14, 29}, two on general heart failure{5, 10}, three on heart failure with reduced ejection fraction (HFrEF) {17, 32}, and one on univentricular heart disease {16}. Four studies targeted the hematologic system: two focused on myeloid leukemia {20, 26}, one on aplastic anemia {33}, and one on myeloma {11}. One study focused on the lymphatic system, with particular emphasis on non-Hodgkin lymphoma {12}. The remaining one study focused on the peripheral vascular system, with an emphasis on critical limb ischemia {25}.

Moreover, three studies targeted the immune system, particularly focusing on graft-versus-host disease (GVHD) {1, 15, 23}. Two of them were specifically designed to address steroid-resistant acute GVHD {1, 23}. Two studies focused on the respiratory system, which plays a role in the pathogenesis of SARS-CoV-2 infection (COVID-19) {4, 6}. Ultimately, one study focused on the endocrine system, targeting type 1 diabetes {28}; another on the reproductive system, targeting gynecological cancer {33}; and the other on the integumentary system, targeting atopic dermatitis {7}.

Overall, the most prevalent conditions targeted by iPSC treatment studies were those affecting the cardiovascular and nervous systems. These findings indicate that the majority of iPSC-based therapies are concentrated on disorders associated with the systems that are commonly regarded as vital and challenging to address using conventional therapeutic modalities. This aligns with the hypothesis that iPSCs are a revolutionary and transformative resource in the field of regenerative medicine. It also highlights the importance of ensuring the safety of iPSC-based therapeutic products.

#### Comparative analysis of iPSC treatment studies by country

**Fig. 8:**
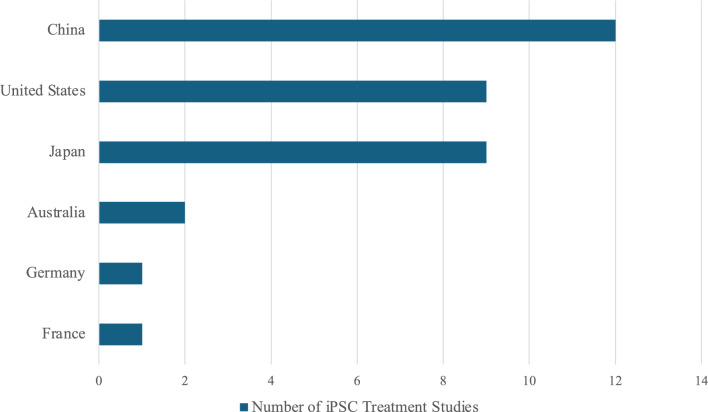
Number of iPSC treatment studies by country. The chart shows the number iPSC treatment studies by the country it was conducted in. This corresponds to the 35 treatment studies indicated in the top box in the rightmost column in Fig. 6.

Subsequently, the studies were classified by country to identify which countries are leading in the development of iPSC-based therapies. Figure 8 shows the number of iPSC treatment studies conducted in each country. While numerous countries are engaged in the development of stem cell treatments, as illustrated in Fig. 5, only seven countries have successfully conducted clinical studies on iPSC-based therapies. Specifically, China has spearheaded the field with 12 studies; all of them were conducted after 2021, and nine of them were initiated within the past two years. This corroborates the findings presented in Fig. 5, which illustrate that China holds a leading position in stem cell therapy research. Also, this suggests that China has recently dedicated a lot of effort to the research and development of iPSC-based therapies.

The United States and Japan were the next most active countries, with nine studies each. As of 2018, both countries are at the vanguard of clinical studies using iPSC-based treatments. The difference between the two is that the United States has maintained a consistent presence in clinical studies from 2019 to 2024, whereas the most recent clinical study in Japan commenced in 2022 (Table 4). In Japan, the total number of stem cell therapy-related clinical studies was 121, which represents less than one-tenth of the 1,992 studies done in the United States. Consequently, Japan is not considered a superpower in stem cell research (Fig. 5). Conversely, Japan has made significant investments in the development of iPSC-based treatments to achieve a level of clinical studies on par with that of the United States.

To date, Germany and France are the only countries in the EU to have carried out iPSC treatment studies. Nevertheless, only a single study has been conducted in each country. This contrasts with the active stem cell research landscape in Europe; Germany, France, Spain, and Italy have reported relatively high numbers of clinical studies on stem cell therapies (Fig. 5). While being active in stem cell research, these countries are lagging behind in the development of treatments specifically derived from iPSCs. This reflects the fact that the EU enforces a more rigorous set of regulations on the research and development of ATMPs, which include iPSC-based therapies.

Switzerland and South Korea fall behind in the number of iPSC treatment studies, with no studies having been conducted in the two countries. Both countries have stringent laws on stem cell research, similar to that of the EU. Switzerland, for instance, implements regulations almost consistent with that of the EU in most stages of development. Regulations in South Korea are also relatively strict; the Bioethics and Safety Act permits clinical trials only when they aim to treat hereditary diseases or when no other treatment options are available for the condition [39]. At the same time, the lack of iPSC treatment studies in South Korea can be attributed to the greater focus on stem cell research and therapy utilizing mesenchymal stem cells (MSCs) and umbilical cord blood stem cells (UCBSCs). To be specific, as of 2023, nine out of twelve approved MSC-based therapies were developed in Asia, and the majority of them were from South Korea [83].

**Fig. 9:**
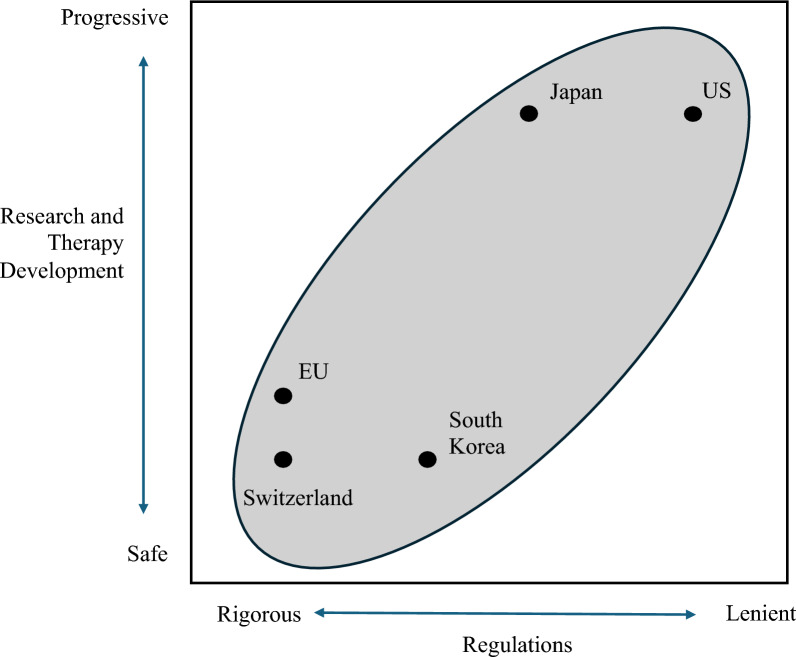
Global tendencies in regulations and development of iPSC-based treatments. The figure illustrates the regulatory trends and advancements in iPSC-based treatments across different countries. The x-axis represents how rigorous regulations are. The y-axis represents how active research on iPSC-based treatments is in each country; the number of iPSC treatment studies was used as a proxy for this measure. A positive correlation shown in the graph strongly supports the fact that less rigorous regulations facilitate research and development of stem cell therapies.

Notably, the number of clinical studies on iPSC-based treatments in the United States and Japan is significantly higher than that in the EU, Switzerland, and South Korea. The discrepancy can be attributed to the progressive and open nature of the regulatory framework governing stem cell therapies in the United States and Japan, which provides a conducive environment for the development of cutting-edge advanced therapeutic modalities, including those involving iPSCs. Lenient regulations reduce hurdles that researchers need to go through in order to complete the development of stem cell therapies. Figure 9 presents the regulatory trends and advancements in iPSC-based treatments across different countries. The x-axis represents how rigorous regulations are, whereas the y-axis represents the number of iPSC treatment studies, which can be used as a proxy for how active research on iPSC-based therapies is in each country. The graph shows a positive correlation between the leniency of the regulation and the number of studies. This further corroborates the hypothesis that less rigorous regulations encourage research and development of stem cell therapies.

However, regulations play an important role in ensuring the safety and efficacy of stem cell therapies. For instance, authorities require researchers to acquire manufacturing licenses before clinical trial and market placement because it gives them better control over the quality of medicinal products during the respective phases of development. This means that loosening up the regulations to be more lenient might come at a price of partially giving up the safety guarantees on stem cell therapies. Hence, well-balanced regulation is important in reducing the associated risks and encouraging the development of new therapeutic modalities. In addition, regulatory science enables regulators to find the right balance between these two values without necessarily trading off one for the other. Regulatory science allows authorities to apply a flexible set of rules to different research projects more on a case-by-case basis, while still upholding a consistent standard for the quality and the safety of stem cell therapies.

**Fig. 10:**
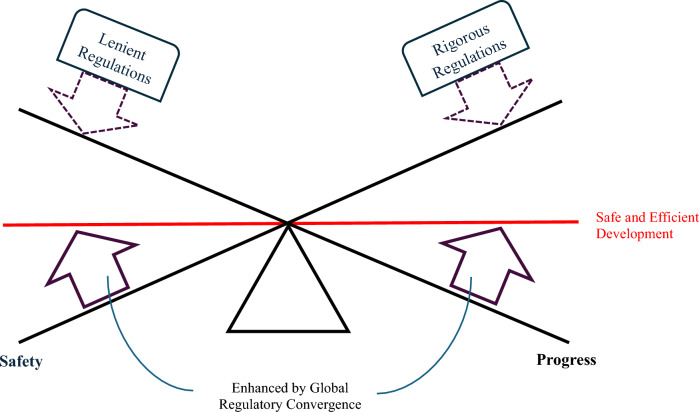
Balancing Safety and Progress in Stem Cell Research and Therapy. The figure illustrates the importance of finding the correct balance between lenient and rigorous modes of regulations in order to achieve both safety and progress in stem cell therapies. It indicates that higher focus on lenient regulations enhance the progress in stem cell research and therapy, while more rigorous regulations improve safety of such therapies. Also, it shows the role that global regulatory convergence can play in reinforcing the move towards safe and efficient development of stem cell therapies.

As previously discussed, countries in the five key regions are on different ends of the policy spectrum when it comes to regulating stem cell therapies. In this context, global regulatory convergence towards a well-balanced approach is imperative, because it improves the applicability and scalability of stem cell therapies. A consistent regulatory landscape facilitates the cross-pollination of research efforts between countries, because research will adhere to a similar set of standards. Additionally, international convergence helps stem cell therapies developed in one region to be market placed in another regime, making them more scalable. Figure 10 illustrates the importance of finding the correct balance between lenient and rigorous modes of regulations in order to achieve both safety and progress in stem cell therapies. Also, it shows the role that global regulatory convergence can play in reinforcing the move towards safe and efficient development of stem cell therapies.

## Conclusions

This study highlights the significant impact of regulatory frameworks on the development of stem cell therapies. For this purpose, a comparative analysis of global regulations and studies on stem cell therapies was conducted.

Regulatory frameworks for stem cell-based medicinal products are categorized into three layers: legislation, executive orders, and guidelines. The study focuses on the legislation layer because while guidelines offer the most practical and specific guidance, there is little variation across countries in terms of scope and substance. The role of the regulatory authorities in interpreting and enforcing such guidelines is important, which indicates that the laws that designate such agencies should be the subject of analysis. According to the investigation, the EU and Switzerland impose relatively rigorous regulations. For example, the EU requires a manufacturing license for market placement and follows the prior authorization model for clinical trials. Such regulations can ensure safety but they may slow down innovation. In contrast, the United States enforces less stringent regulations and relies heavily on regulatory science, which promotes faster progress in the development of stem cell therapies. To be specific, it adopts a prior notification model and permits provisional authorization.

Japan and South Korea strike a balance, incorporating practices from both regimes. For instance, South Korea does not require a manufacturing license for clinical trials but follows the prior authorization model; the opposite is true in Japan, where manufacturing license is required for both clinical trial and market placement, but a prior notification model is used. Between the two countries, South Korea tends to impose more stringent regulations than Japan. For example, South Korea strictly bans iPSC-based germline therapies, just like the EU. Japan adopts a more flexible regulatory approach akin to that of the United States, such as permitting germline editing for research purposes.

Further analysis was done on therapeutic studies involving iPSC-based clinical trials. First, the clinical trials were categorized based on the conditions they were targeting. The result revealed that clinical trials largely target diseases affecting the cardiovascular and nervous systems, both of which are considered vital. Hence, ensuring the safety of iPSC-based therapeutic products is particularly important, as they may pose a great risk to patients. Second, iPSC-based clinical studies were aggregated based on the country it was conducted in. Countries where regulation on stem cell research was relatively lenient, such as the United States and Japan, had the largest numbers of clinical trials. Germany, France, Switzerland, and South Korea, all of which have relatively rigorous regulations on stem cell research, trailed behind. This finding indicates that lenient regulations can lead to more rapid advances of iPSC-based therapies.

Therefore, it is imperative to take a well-balanced approach when regulating stem cell therapies, in order to facilitate technological progress while minimizing the associated risks. Although a rigorous set of regulations better asserts the safety of such treatments, they might hinder research and development, and vice versa. In addition, regulatory agencies around the world should coordinate to achieve international convergence towards a balanced regulatory scheme. Global regulatory convergence is necessary to enhance the applicability and scalability of stem cell therapies, as it makes it easier for treatment developed in one regime to be placed in another market. A consistent standard across the board also provides researchers a clearer guidance and facilitates international collaboration during the development process. Towards this end, each country should adopt global standards and, at the same time, impose a localized version of regulations based on its cultural context and circumstances.

## Data Availability

All data (or sources thereof) relevant to this study are included in the article, and further inquiries can be directed to the corresponding author.
